# Spectator medicine in the men’s international Ice Hockey World Championships in 2022 and 2023

**DOI:** 10.1186/s12913-025-13504-z

**Published:** 2025-10-03

**Authors:** Ville Bister, Kerttu J. Toivo, Timo H. Hänninen, Collins Hart, Markku P. Tuominen

**Affiliations:** 1 Helsinki University Hospital Trauma Unit, Helsinki, Finland; 2 Department of Surgery, Clinicum, Faculty of Medicine, University of Helsinki, Helsinki, Finland; 3 Tampere Research Centre of Sports Medicine, UKK Institute, Tampere, Finland; 4 Medisport Ltd, Tampere, Finland

**Keywords:** Ice hockey, Spectator medicine, Major event

## Abstract

**Introduction:**

Sports events attract large crowds, necessitating the provision of medical services. Medical issues at these events can include acute injuries, such as falls or incidents related to the sport being played, as well as the worsening of pre-existing health conditions.

**Study objective:**

The aim of this descriptive study is to report on the extent and type of spectator medicine services needed during major sporting events in ice hockey and what factors affected the use of these services.

**Methods:**

Data was collected from two top-level men’s world championship tournaments in Finland consisting of 98 games with a total of over 630,000 spectators. Trained nurses working in pairs reported the type, treatment and time of day of spectator medicine services provided at the tournaments, as well as the number of patients requiring treatment outside the arena.

**Results:**

The average number of health care contacts per game was 3.9. The number of health care contacts increased in concordance with audience size, but when adjusted for audience size there were fewer contacts in host team Finland’s games (0.55, 95% CI 0.3–0.9). 30% of the contacts were due to skin conditions, followed by musculoskeletal conditions and headaches. A total of 25 patients required treatment outside the arena, with 9 requiring an ambulance transfer.

**Conclusion:**

The preparatory measures and the number of medical staff present were at times abundant, but nevertheless sufficient for the needs of the spectators. This resulted in only a small minority of patients needing to be transferred for further treatment outside the arena. For future events, we recommend there is at least one nurse for every 1000 expected spectators. Additionally, nurses should work in pairs to be able to effectively initiate treatment for severe medical conditions.

**Clinical trial number:**

Not applicable.

## Introduction

The International Ice Hockey Federation (IIHF), together with national ice hockey associations, organises world championship tournaments every year [1]. Ice hockey is a very popular sport in Finland, with 68,417 registered players or officials in 2023 − 24 [2]. The Ice Hockey World Championships gathers large numbers of spectators to multiple indoors events. The first world championship tournament in Finland was held in Tampere in 1965, followed by tournaments in 1974, 1982, 1991, 1997 and 2003 in the varying host cities of Helsinki, Tampere and Turku [3]. In 2012, the world championship tournament was shared and held in both Sweden and Finland, with a total audience of 451,054 (an average of 7084 per game). The tournament was also shared between Finland and Sweden in 2013, with a total audience of 427,818 (an average of 6685 per game). In both the 2012 and 2013 tournaments, the host cities were Helsinki, Finland and Stockholm, Sweden.

Previous studies of outdoor mass gathering events have shown a patient presentation rate of 2.63–20 per 10,000, including a considerable proportion of environment-induced problems; but the majority of cases (91.5-95.97%) have been mild in severity [4–7]. There have been several and more thorough studies on ice hockey players’ injuries and safety [8–12]but studies on the spectators’ side are scarce and limited [13–16]. Large scale sporting events held in May in Finland include the possibility of dehydration due to the warming weather conditions in both indoor and outdoor areas of the arena, combined with abundant alcohol consumption [17–20]. Access to e.g., mineral water for rehydration should be available to help prevent or alleviate symptoms such as headaches and nausea.

Effective planning is crucial [21]. By understanding the demand for medical services at a large indoor sporting event, resources can be more effectively allocated and organisers can be better prepared in the future. The objective of this study was to find out the extent and type of spectator medicine services needed during the 2.5-week IIHF Men’s Ice Hockey World Championship tournaments held in 2022 and 2023, and what factors affected the use of these services. During both tournaments, one half of the quarterfinals and all of the medal games were played in Tampere, Finland. The hypothesis is that during host team Finlands games, the health care contact rates will be higher compared to other games.

## Methods

### Preparations

The 2022 Men’s Ice Hockey World Championship tournament was held in Tampere (Nokia Arena, max. capacity of 13,455 spectators) and Helsinki (Helsinki Ice Hall, max. capacity of 8200 spectators), Finland, and the 2023 tournament in Tampere and Riga, Latvia. This study will present the model and results of the spectator medicine unit at the world championship tournament from the venues in Finland. Preparation for the 2022 tournament began in spring 2021 by forming the event chief medical officer team to design and lead medical services for both players and spectators. Volunteer registration was opened soon after. The medical team responsible for first aid services for spectators consisted mainly of trained nurses (paramedic, nurse, practical nurse). Both Tampere and Helsinki had their own medical teams, which were formed and trained in a similar manner. However, in Tampere (which was the main venue during both years), half of the volunteer nurses were members of an organisation named Tamrescue; they had experience of working as medical staff in sporting events and were familiar with the arena. The spectator medicine unit was strictly separated from players’ health care due to accreditation procedures, however the chief medical officers provided support to both the player and spectator sides when needed.

The task-targeted training of the spectator medicine unit consisted of team building, general information, radio phone training, familiarisation with the arena’s facilities and hands-on training (e.g., evacuation of a prone patient from the stands). Altogether, five training sessions (three in 2022, and two in 2023), each lasting for 2–3 h, were organised and quick recap of the tasks was arranged before everyone’s first shift.

The spectator medicine staff for each game consisted of consulting chief medical officer and 6–10 trained nurses who started their shift when the arena opened (one hour before the game) and ended when the spectators had left the arena (30–60 min after the game). Game times including pre- and post-work, and their proportions are presented in Table 1. The two-nurse teams were positioned as evenly as possible throughout the arena. One pair (the senior nurse and an assistant) was stationed in the designated first aid room while other pairs circulated the arena on different sides and at different levels, two altogether. While the match was being played, the pairs located in the stands to have clear visibility to most of the spectators and were in close collaboration with the arena’s security staff who were usually the first arena personnel to be contacted if any medical issues occurred. All pairs had radio phones for communication. In Tampere, one of the nurses in each pair was a member of Tamrescue and therefore familiar with the arena. After each game, the spectator medicine staff moved to the lobby to ensure a safe exit from the arena. Every evening after the last game, a motivational and instructional message was sent to the whole spectator medicine unit via mobile phones.

**Table 1:** The mean number of health care contacts, number of patients requiring treatment outside the arena, and number of patients requiring transfer to hospital by ambulance per 1000 spectators at different times of the day

Time of day		Day (11 am-3 pm)	Early evening (3 pm-7 pm)	Late evening (7 pm-11 pm)
Attendance at the game (avg.)		5738 ± 2233	6282 ± 3182	6831 ± 3609
Health care contacts (all, n)		41	187	155
Games (all, n)		12	43	43
Health care contacts	per 1000 spectators: mean, (95%CI), p-value*	0.62, (0.42–0.92), *p* = 0.74	0.70, (0.57–0.86)	0.52, (0.42–0.64) *p* = 0.05
Required treatment outside arena		0.01, (0.00-0.07), *p* = 0.49	0.02, (0.01–0.05)	0.06, (0.04–0.09), *p* = 0.013*
Required ambulance transfer		0.00, (0.00–1.00), *p* = 0.17	0.0, (0.00-0.03)	0.02, (0.01–0.06), *p* = 0.18

*Statistical significance indicated with p-value comparing with the early evening game, *p* < 0.05

The spectator medicine nurses were allowed to make independent decisions in treating patients. If a doctor’s assistance was desired, the event chief medical officer was contacted via radio phone or mobile phone. Nurses carried basic first aid supplies along with them and distributed basic medications (paracetamol, ibuprofen, cetirizine) that are prescription-free in Finland, after confirming possible allergies and contraindications. These medical services were cost-free to the spectators. Defibrillators were available both at fixed points within the arena and with one of the pairs circulating the arena. Most of the volunteers were experienced emergency room nurses. If the diagnostic procedure or further treatment was needed, the transporting ambulance was called via general emergency number 112, or the spectator was advised to continue to the nearest health care unit for further evaluation, if the situation was deemed “mild” and the person was in full understanding of the situation.

According to IIHF regulations, a separate medical service with its own first aid room and staff must be provided for spectators (https://blob.iihf.com/iihf-media/iihfmvc/media/downloads/regulations/2022/2022_iihf_medical_regulations.pdf*).* An emergency action plan for spectator medicine was compiled according to Finnish regulations and was sent to and reviewed by the local emergency medicine department. Predetermined meeting points were established in case of a severe multi-patient situation and medical staff were informed of where ambulances would arrive in case of an emergency on the spectators’ side.

### Data source

The research data collection chart was planned, designed, and finalised before the tournament. The idea was to create a “simple to use” form which gathers the right amount of information while minimising the possibility of lacking information. The data collected from tournaments organised in Finland included the date, location of the pair in the arena, time of day, type of contact (none/injury/illness), reason for contact, note if other than spectator, procedure/medication, and any transportation used (destination, means). This information was documented after the patient was treated. All charts were collected and reviewed daily by the event chief medical officer. In addition, if any suspicion of further medical problems, the nurses wrote a more specific report which is not included in this material. Data from Riga, Latvia was not collected, as described.

### Data handling and statistical analysis

The data from the hand-filled charts was transferred to a Microsoft Excel table by VB and CH. Further analysis of the distribution of contacts was carried out by KT. The number of spectators present at each game was gathered from the IIHF Competition Schedule and each game’s Game Centre (https://www.iihf.com/en/events/2022/wm/schedule and https://www.iihf.com/en/events/2023/wm/schedule) by VB. The classification of healthcare contacts was primarily organ-specific, but we added the number of injuries because they are largely preventable and occur frequently.

Statistical analysis was performed using Microsoft Excel and SPSS version 29. A negative binomial regression model was used to analyse the effect of the host team playing on the number of contacts, and the model was adjusted according to the size of the audience. For ambulance transfers, logistic regression was used due to the small number of cases. The data has been normalized per 1000 spectators.

### Ethical considerations

The data has been collected in the context of health care contacts which have been conducted according to good clinical practice. No personalising patient register has been created. This is a descriptive study in which possible patients and various injuries and illnesses are reported by groups and trends. Approval for the study (FIHA 18/05/23) was obtained from the organising committee. The University of Helsinki Research Ethics Committee of the Faculty of Medicine was consulted and since research material doesn’t consist of any identifying patient information, further processing was not required. The study has been carried through in accordance with the Declaration of Helsinki (https://www.wma.net/policies-post/wma-declaration-of-helsinki/).

## Results

In 2022, the total attendance of all 64 games played in both cities was 356,955. In 2023, 34 games were played in Tampere with a total attendance of 275,230 people. The number of patient contacts, spectators per patient, and spectators per hour (game event duration is 4 h) are presented in Table 2. It is notable that the host team, Finland, played all their games in both years in Tampere, which is clearly reflected in the size of the audience.

**Table 2:** The number of games, attendance, patient contacts, spectators per patient, and spectators per hour at different venues each year

Tampere (2022 | 2023)	34	34	Helsinki (2022)	30
Attendance	260,540	275,230	Attendance	96,505
Patient contacts	184	152	Patient contacts	47
Per 10,000 spectators	7.06	5.5	Per 10,000 spectators	4.87
Spectators per patient	1415	1811	Spectators per patient	2053
Patients per hour	1.35	1.12	Patients per hour	0.39

### Factors affecting the number of health care contacts

**Fig. 1:**
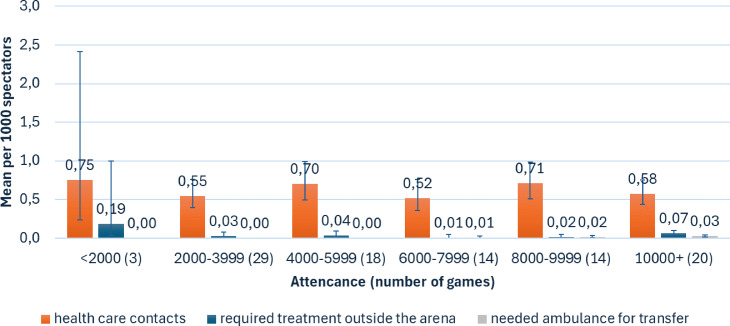
The bars show the mean number of health care contacts (orange) with lines depicting 95% CI, the number of patients requiring treatment outside the arena (blue), and the number of patients requiring transfer to hospital by ambulance (grey) per 1000 spectators at the Men’s Ice Hockey World Championship tournaments in Finland in 2022–2023. The size of audience is shown on the horizontal axis, with the number of games with the given audience in parenthesis

The mean number of all spectator health care contacts per 1000 spectators ranged from 0.52 to 0.75 and did not increase significantly with increasing audience size. The mean number of patients requiring treatment outside the arena was significantly higher when the audience size was > 10 000 compared with 6000–7999 (0.07 vs. 0.01, *p* = 0.023). Also, the mean number of patients requiring ambulance transfer was significantly higher with an audience of more than 10 000 people (0.03) compared to an audience of 4000–5999 (0.00), 2000–3999 (0.00), and < 2000 (0.00) (*p* = 0.000). (Fig. 1). The maximum audience size in these tournaments was 12,056. Host team Finland played a total of 18 games, all of which gathered over 10,000 spectators; i.e., 90% of the games with over 10,000 spectators. The other two games were the bronze medal and gold medal games in 2023.

When the host team, Finland, was playing, the number of spectator medicine contacts was 1.8 times higher than in other games (95% CI 1.2–2.6). When team Finland was playing, there were on average 2.7 more contacts than in games where team Finland was not playing (*p* = 0.016). The number of patients requiring treatment outside the arena was 5.7 times higher (95% CI 2.4–13.2) when Finland was playing than in other games. The mean number of spectators requiring treatment outside the arena was 0.78 when team Finland was playing and 0.14 when they were not (*p* = 0.007).

When adjusting the model for audience size, the number of first aid contacts during team Finland’s games was almost half that of other games (0.55, 95% CI 0.3–0.9). When Finland was playing and the model was adjusted for audience size, the mean number of contacts was 2.1 vs. 3.8 when Finland was not playing (*p* = 0.002). When adjusting for audience size, the number of patients requiring treatment outside the arena when Finland was playing was 4.5 times higher than when other teams were playing, but this difference was not statistically significant (95% CI 0.8–25). Every thousand additional spectators in the audience increased the number of spectator medicine contacts by 16.4% (11.4–21.6). Furthermore, the frequency of an ambulance being required was 5.3 times higher when host team Finland was playing (*p* = 0.03); this was explained by the larger audiences at these games, though this result was not statistically significant (*p* = 0.19, 95% CI 0.01–2.4).

The time of day did not have a significant effect on the number of all health care contacts, but more patients needed to be transferred for treatment outside the arena at late evening games compared to early evening (Table 1). There were slightly more health care contacts during early evening games, but the difference in the number of contacts during different times of the day was not statistically significant, even when adjusting the statistical model for audience size. The number of health care contacts per game ranged from 0 to 15, and a total of nine games did not have any spectator medicine contacts (9% of the games). There was an increase in average number of patients and statistical significance when comparing weekdays (Monday to Thursday) to weekends (Friday to Sunday), 3.0 vs. 4.7 (*p* = 0.008).

### Reasons for spectator medicine contacts

There were altogether 184 traumatic cases and 199 other medical issues. The majority, 70% of the patients, were contacted and treated in the first aid room, 15% at lower level of the arena, and 15% in the higher level of the arena, respectively. 30% of spectator medicine contacts were due to skin issues (wounds, blisters and nail problems) and the second most common presentations were injuries and other musculoskeletal pain conditions at 22%. Equally common were headaches (including migraines), also at 22% (Table 3). 11% of spectator health care contacts were classified as check-ups after tripping/falling/hitting an object; nine of these were cases of being hit by the puck. Conditions are only mentioned in Table 3 if ten or more patients had the same reason for contact. It was believed that alcohol consumption might play a notable role in certain healthcare contacts, however during the tournaments there were just two cases where alcohol overconsumption was the main reason for the contact. An ambulance was required at a total of 8 out of 98 games. Fortunately, potential life-threatening conditions, such as chest pain and dyspnoea (*n* = 1) were rare.

**Table 3:** The most common reasons for spectator health care contacts at the men’s ice hockey world championship tournaments in Finland in 2022–2023. The number of health care contacts is shown after the reason for each condition and the number in brackets represents the number of cases transferred outside the arena for treatment

Reasons for health care contacts and total number of patients (transferred to hospital or medical clinic outside the arena)
1. skin problems (wounds, blisters, nail problems) 114 (7)
2. musculoskeletal complaints including musculoskeletal injuries and pain 86 (6)
3. headaches or migraines 83 (1)
4. cases of falling on stairs and flat ground, being crushed, or being hit by an object 44 (9)
5. malaise, fatigue, weakness, dizziness, or collapse 27 (1)
6. head injuries 16 (3)
7. allergic reaction 13 (0)
8. gastrointestinal symptoms 13 (1)
Altogether 25 patients were transferred to a health care unit outside the arena (9 needed ambulance for transfer). Some of the reasons were present in various combinations.

## Discussion

The total number of spectators seeking medical attention was 383 in a total of 98 games. During each game event there were 0.98 patients per hour, and 93.5% of patients were treated completely within the facilities, i.e., without the need to transfer the patient to a hospital or medical clinic outside the arena. The in-game spectator medicine unit consisted of 6–10 trained nurses and a physician who was available for consultation. Thus, the correct approach was taken regarding having sufficient professional medical staff in the field to meet patients’ urgent medical needs. There was approximately one patient per 1650 spectators; in future, therefore, events should plan to have at least one nurse per 1000 expected spectators. However, it should also be borne in mind that nurses at events should work in pairs in order to be able to properly initiate treatment for more severe medical conditions such as cardiac arrest. It is also essential that nurses are able to take breaks. Furthermore, whether a given game is a host team game or a medal game should be taken into consideration when determining the number of medical staff required, because these types of events appear to gather the most spectators. In this case all except one of the host team games and medal games gathered over 10,000 spectators. Quite surprisingly, when adjusting for audience size, the frequency of patient contacts when host team Finland was playing was lower than at games where they were not. There is a possibility, that this could be explained by the presence of a larger number of foreign spectators at games where the host team was not playing, since this group of people may be more likely to seek medical help at the arena compared to locals (who know how to access medical treatment in their own country). The “weekend-effect” has been shown in the increasing number of traumatic cases [20]. Our results showed statistically significant weekend-effect in overall number but not in increasing trauma cases.

Mass gatherings often involve the use of alcohol, which increases the risk of mild to severe injuries both inside and outside the event area [21–23]. In our results there were only two cases of alcohol overuse, which is markedly lower than it might have been. Voluntary trained health care professionals are needed to make tournament attendance safer for spectators and lower the stress on the public health care system. It has been recognised that during mass gathering events there is an increased workload for emergency medical services, not only inside the event area but also on the outskirts of it, as presented by Koski and colleagues in 2022 [23]. In order to improve public health care planning for mass gathering event periods, it has been suggested that a comprehensive collection of hospital data should also be included in forthcoming studies [24]. Melegari and colleagues, in describing the medical management of a major planned event which more than doubled the population of the host city, highlighted the importance of synergistic collaboration with local hospitals [21]. The findings of our study do not encompass all of the stress imposed on the public health care system during the championship tournament. This issue should be considered when preparing for the next large-scale ice hockey event in Finland.

Goldberg and colleagues calculated the rate of transportation to a hospital as 1.6 per 10,000 [5]. In our study, this should have translated to 57 patients, but our actual number was markedly lower: 25 patients, of which 9 were transferred by ambulance, giving a rate of 0.4 per 10,000. With a total of 632,185 spectators, this is an exceptionally low number; but, in this study, it may be explained by nurses treating most cases inside the arena, leading to fewer cases of transportation elsewhere. In this type of arrangement, a patient needed to be transferred outside the arena approximately once every 1–2 days, and an ambulance was required only once every 3–4 days, and only once the case was considered a life-threatening emergency. This has led us to think that if the municipal emergency medical response time is adequate (e.g. 5 to 15 min), is the need to retain an ambulance on-site truly necessary for waiting for the load-and-go situation? Off course in multipatient situations it may have its advantages.

There were a total of nine events in which a spectator was injured due to being hit by the puck. When planning spectator medical services, sport-specific risks need to be evaluated and planned for. In these tournaments, the arena’s security staff were instructed to check whether there was a need for medical assistance in cases where the puck flew into the crowd.

A European consensus document was published in 2011 regarding cardiovascular safety, with recommendations for preparing a medical action plan and the number of medical staff to have on site [25]. Preparations were made for single-patient worst case scenarios such as resuscitation in the stands, although mostly common medical issues, i.e., bruises, wounds, headaches, etc., were expected. The results of this study are in line with earlier reports showing that a variety of skin problems form the majority of patient consultations and treatments [24] and therefore materials for wound care should be kept well-stocked. As well as spectators, some other volunteers and arena staff in the spectator area also used the medical services for minor complaints, if this helped them complete their shift. In addition to the issues reported in this manuscript, and rare and severe cardiac issues, it is important to consider the risk of terrorism and violence at major sporting events. The possibility of highly transmissible pathogens should also be considered.

### Limitations

A significant limitation of this study is that the results do not include those who may have had an injury or illness and did not seek evaluation at the arena. With the aim of making it as easy as possible to contact medical services, the medical room was clearly indicated and medical staff were easily recognisable. Patients age, gender, nationality, and alcometer count would have added more value to further analysis. Also, hospital records would have probably shown more impact on the public health care, and this should be taken into account in future tournaments. The sample size is two tournaments, which is limited, and more would be better. Some national differences exist, therefore presenting the results in Finnish context is justified. The focus here was an international one discipline (ice-hockey) tournament in one country, with the most spectators being Finns, though including also fans from other countries, these results are not strictly generalisable.

## Conclusions

We wanted to find out what is the variety and proportion of the healthcare contacts, and now we know. The preparatory measures and the number of medical staff at these events were sufficient for the needs of the spectators (in every game 6 to 10 trained nurses with consulting physician available), resulting in only a small minority of patients needed to be transferred for further treatment outside the arena. Surprisingly host team Finland games had lower rate of contacts when adjustments for attendance was counted but altogether increasing spectator count increases the healthcare contacts to be dealt with. In future top level ice hockey tournaments, we recommend there is at least one nurse for every 1000 expected spectators; however, nurses should work in pairs to be able to effectively initiate treatment for severe medical conditions, as a team which supports each other.

## Data Availability

Data available on reasonable request from authors.

## Declarations

### Use of AI technology

AI technology was not used to generate text for the manuscript OR to aid in making minor improvements to the text.

## Abbreviation

IIHF: International Ice Hockey Federation

## References

[1] iihf.com. https://www.iihf.com/en/tournaments

[2] The Finnish Ice Hockey Association Annual Report. 2023–24. https://www.finhockey.fi/index.php/info/vuosikertomus

[3] Finnish ice hockey history. https://www.leijonat.fi/index.php/sarjat/suomi-sarja/item/13512-historia

[4] De Lorenzo RA. Mass gathering medicine: A review. Prehosp Disaster Med. 1997;12(1):68–72. 10.1017/S1049023X00037250. 10166378 10.1017/s1049023x00037250

[5] Goldberg SA, Maggin J, Molloy MS, et al. The Gillette stadium experience: A retrospective review of mass gathering events from 2010 to 2015. Disaster Med Public Health Prep. 2018;12(6):752–8. 10.1017/dmp.2018.7. 29552999 10.1017/dmp.2018.7

[6] Locoh-Donou S, Guofen Y, Welcher M, Berry T, O’Connor RE, Brady WJ. Mass-gathering medicine: a descriptive analysis of a range of mass-gathering event types. Am J Emerg Med. 2013;31(5):843–6. 10.1016/j.ajem.2013.01.016. 23453125 10.1016/j.ajem.2013.01.016

[7] Tajima T, Takazawa Y, Yamada M, et al. Spectator medicine at an international mega sports event: rugby world cup 2019 in Japan. Environ Health Prev Med. 2020;25(1):72. 10.1186/s12199-020-00914-0. 33234126 10.1186/s12199-020-00914-0 PMC7684143

[8] Soligard T, Palmer D, Steffen K, et al. Sports injury and illness incidence in the PyeongChang 2018 olympic winter games: a prospective study of 2914 athletes from 92 countries. Br J Sports Med. 2019;53(17):1085–92. 10.1136/bjsports-2018-100236. 31235615 10.1136/bjsports-2018-100236

[9] Soligard T, Steffen K, Palmer-Green D, et al. Sports injuries and illnesses in the Sochi 2014 olympic winter games. Br J Sports Med. 2015;49(7):441–7. 10.1136/bjsports-2014-094538. 25631542 10.1136/bjsports-2014-094538

[10] Tuominen M, Stuart MJ, Aubry M, Kannus P, Parkkari J. Injuries in world junior ice hockey championships between 2006 and 2015. Br J Sports Med. 2017;51(1):36–43. 10.1136/bjsports-2016-095992. 27281776 10.1136/bjsports-2016-095992

[11] Tuominen M, Stuart MJ, Aubry M, Kannus P, Parkkari J. Injuries in men’s international ice hockey: a 7-year study of the international ice hockey federation adult world championship tournaments and olympic winter games. Br J Sports Med. 2015;49(1):30–6. 10.1136/bjsports-2014-093688. 25293341 10.1136/bjsports-2014-093688 PMC4316846

[12] Tuominen M, Hänninen T, Parkkari J, et al. Concussion in the international ice hockey world championships and olympic winter games between 2006 and 2015. Br J Sports Med. 2017;51(4):244–52. 10.1136/bjsports-2016-097119. 28148512 10.1136/bjsports-2016-097119

[13] Myles WM, Dickinson JD, LaRoche GR. Ice hockey and spectators’ eye injuries. N Engl J Med. 1993;329(5):364–364. 10.1056/NEJM199307293290520. 8321276 10.1056/NEJM199307293290520

[14] Piira OP, Miettinen JA, Hautala AJ, Huikuri HV, Tulppo MP. Physiological responses to emotional excitement in healthy subjects and patients with coronary artery disease. Auton Neurosci. 2013;177(2):280–5. 10.1016/j.autneu.2013.06.001. 23916871 10.1016/j.autneu.2013.06.001

[15] Read C, Beaumont C, Isbell J, et al. Spectator injuries in sports. J Sports Med Phys Fit. 2019;59(3). 10.23736/S0022-4707.18.09146-6. 10.23736/S0022-4707.18.09146-6 30411604

[16] Milsten A, Ness J. Hockey puck strike rates and injuries at National hockey league games: A retrospective analysis of data from six seasons. Prehosp Disaster Med. 2022;37(3):397–400. 10.1017/S1049023X22000619. 35435157 10.1017/S1049023X22000619

[17] Yamamoto R, Maeshima K, Asakawa S, et al. Development of On-Site medical system for Mass-Gathering events during TOKYO 2020: vulnerability analysis using healthcare failure mode and effect analysis. Disaster Med Public Health Prep. 2023;17:e66. 10.1017/dmp.2021.329. 10.1017/dmp.2021.329 34847980

[18] Warpenius K, Mäkelä P. The Finnish drinking habits survey: implications for alcohol policy and prevention. Nordic Stud Alcohol Drugs. 2020;37(6):619–31. 10.1177/1455072520954328. 10.1177/1455072520954328 PMC8899282 35308649

[19] Mäkelä P, Warpenius K. Night-time is the right time? Late‐night drinking and assaults in Finnish public and private settings. Drug Alcohol Rev. 2020;39(4):321–9. 10.1111/dar.13068. 32291837 10.1111/dar.13068

[20] Mäkelä P, Martikainen P, Nihtilä E. Temporal variation in deaths related to alcohol intoxication and drinking. Int J Epidemiol. 2005;34(4):765–71. 10.1093/ije/dyi025. 15737967 10.1093/ije/dyi025

[21] Melegari G, Giuliani E, Fornaciari D, Cremonini C, Di Pietro G, Barbieri A. Health care management during a major planned event in Italy. Prehosp Disaster Med. 2022;37(6):847–52. 10.1017/S1049023X22001352. 36189691 10.1017/S1049023X22001352

[22] Ranse J, Hutton A, Keene T, et al. Health service impact from mass gatherings: A systematic literature review. Prehosp Disaster Med. 2017;32(1):71–7. 10.1017/S1049023X16001199. 27938460 10.1017/S1049023X16001199

[23] Koski A, Pappinen J, Kouvonen A, Nordquist H. Preparedness for mass gatherings: rescue and emergency medical services’ workloads during mass gathering events. Scand J Trauma Resusc Emerg Med. 2022;30(1):15. 10.1186/s13049-022-01003-7. 35248139 10.1186/s13049-022-01003-7 PMC8898448

[24] Borjesson M, Serratosa L, Carre F, et al. Consensus document regarding cardiovascular safety at sports arenas: position stand from the European association of cardiovascular prevention and rehabilitation (EACPR), section of sports cardiology. Eur Heart J. 2011;32(17):2119–24. 10.1093/eurheartj/ehr178. 21672932 10.1093/eurheartj/ehr178

[25] Van Remoortel H, Scheers H, Lauwers K, et al. Mass gathering events: a retrospective analysis of the triage categories, type of injury or medical complaint and medical usage rates. Emerg Med J. 2022;39(9):708–11. 10.1136/emermed-2021-211745. 35393345 10.1136/emermed-2021-211745

